# De novo transcriptome assembly, functional annotation and differential gene expression analysis of juvenile and adult *E. fetida*, a model oligochaete used in ecotoxicological studies

**DOI:** 10.1186/s40659-017-0114-y

**Published:** 2017-02-27

**Authors:** Michelle Thunders, Jo Cavanagh, Yinsheng Li

**Affiliations:** 1 College of Health, Massey University, PO Box 756, Wellington, 6140 New Zealand; 2 Landcare Research, PO Box 40, Lincoln, 7640 New Zealand; 3 School of Agriculture and Biology, Shanghai Jiao Tong University, Shanghai, 200240 China

**Keywords:** Earthworm, Trinity, Transcriptome, *E. fetida*, Ecotoxicology, RNA, Sequence, Gene expression

## Abstract

**Background:**

Earthworms are sensitive to toxic chemicals present in the soil and so are useful indicator organisms for soil health. *Eisenia fetida* are commonly used in ecotoxicological studies; therefore the assembly of a baseline transcriptome is important for subsequent analyses exploring the impact of toxin exposure on genome wide gene expression.

**Results:**

This paper reports on the de novo transcriptome assembly of *E. fetida* using Trinity, a freely available software tool. Trinotate was used to carry out functional annotation of the Trinity generated transcriptome file and the transdecoder generated peptide sequence file along with BLASTX, BLASTP and HMMER searches and were loaded into a Sqlite3 database. To identify differentially expressed transcripts; each of the original sequence files were aligned to the de novo assembled transcriptome using Bowtie and then RSEM was used to estimate expression values based on the alignment. EdgeR was used to calculate differential expression between the two conditions, with an FDR corrected P value cut off of 0.001, this returned six significantly differentially expressed genes. Initial BLASTX hits of these putative genes included hits with annelid ferritin and lysozyme proteins, as well as fungal NADH cytochrome b5 reductase and senescence associated proteins. At a cut off of P = 0.01 there were a further 26 differentially expressed genes.

**Conclusion:**

These data have been made publicly available, and to our knowledge represent the most comprehensive available transcriptome for *E. fetida* assembled from RNA sequencing data. This provides important groundwork for subsequent ecotoxicogenomic studies exploring the impact of the environment on global gene expression in *E. fetida* and other earthworm species.

**Electronic supplementary material:**

The online version of this article (doi:10.1186/s40659-017-0114-y) contains supplementary material, which is available to authorized users.

## Background

Earthworms are exposed to environmental contaminants through their intestine, skin, and alimentary and dermal uptake routes and are widely used as a model organism for assessing impact of environmental chemicals and toxins [1–3]. Earthworms are sensitive to soil pollution that arises from intensive use of biocides in agriculture, industrial activity and atmospheric deposition [4] and so are useful bio-indicator organisms (Sivakumar). *Eisenia fetida* is a commonly used test species in ecotoxicological studies [5]. Molecular genetic data are now available on the response of worms to toxin exposure, particularly focusing on the antioxidant and stress protein response to pollutants [6–8]. In order to establish the impact of exogenous toxins on gene expression at the genome level it is first important to assemble a baseline transcriptome for the model organism. Gong et al. produced transcriptome wide oligonucleotide probes for *E. fetida* in their [9] report, comprising 63541 ESTs, but a complete annotated transcriptome for *E. fetida* has not been available thus far for this organism. Many environmental toxins have putative roles in impeding reproduction [10], therefore identifying differentially expressed genes between adult and juvenile *E. fetida* can be useful to identify possible genes associated with sexual maturity and reproduction that may be more susceptible to such toxins.

## Methods

Worms were established in a composting wormery from an initial population of 150 *E. fetida*. A box of worms was bought from Bunnings (Lyall Bay. Wellington, NZ) and grown in a composting wormery supplemented with fruit and vegetable waste for 6 months. Adult worms were selected on the basis of well-developed clitella, present only at sexual maturity; juveniles were selected on the basis of the absence of well-developed clitella. Adult worms were dissected to enhance samples for the reproductive organs including gonadal structures. For each extraction four gonadal segment samples were pooled to maximize RNA content. RNA was extracted from pooled juvenile and fully clitellated adult samples respectively and then sequenced using Illumina HiSeq 2500, sequencing was carried out by New Zealand Genomics Ltd (http://www.nzgenomics.co.nz/). Prior to RNA extraction, worms were removed, washed with RNAse, DNAse free water and then used for total RNA extraction. The total RNA was isolated using the QIAGEN Mini kit and by following the manufacturers protocol. Extra vigilance was taken to minimise degradation of RNA; RNAse zap was used to clean all dissection tools and equipment used in the RNA extraction process. Trimmomatic was used to quality trim reads using the default settings and Trinity v2.1.1, and Edger 3.3 were used for de novo transcriptome assembly,including, identification of candidate coding regions, and differential gene expression analysis as outlined by Haas et al. [11]. Transdecoder v2.01, Trinotate v2.0.2, BLASTX v2.3.1, BLASTP v2.3.0+ and HMMER 3.0 searches were used for functional annotation of the transcriptome produced and to populate an Sqlite database. All bioinformatic analyses were carried out via VPN connection to a remote high speed computer (CPU 16) on the BioIT, NZGL network via a standard PC laptop.

## Results

### De novo transcriptome assembly

Trinity is a freely available software tool used for de novo transcriptome assembly that uses three software modules (Inchworm, Chrysalis and Butterfly) to assemble a de novo transcriptome when no model data is available [12], it does this by first assembling the RNA sequence data into transcripts or ‘genes’, clustering these contigs, constructing de Bruijn graphs and then processing the graphs to report full length transcripts for all alternatively spliced isoforms. The resulting transcriptome file was generated in fasta (.fq) format and was 32 MB in size comprising 74,381 ‘genes’, 90,862 ‘transcripts’ and with a GC percentage of 41.74. The N50 was 339 and 329 for transcripts and genes, respectively, which is the maximum length where at least 50% total assembled sequence resides in contigs of at least that length; further details on the transcriptome are found in Table 1. At a stringency of a minimum 5 TPM there were 21282 estimated ‘genes’.

**Table 1:** Summary of constituent data of Trinity-assembled *E. fetida* transcriptome

Total Trinity ‘genes’	74,381
Total Trinity transcripts	90,862
Percent GC	41.74
Stats based on ALL transcript contigs	
Contig N10	788
Contig N20	586
Contig N30	472
Contig N40	395
Contig N50	339
Median contig length	276
Average contig length	340.18
Total assembled bases	30,909,011
Stats based on ONLY LONGEST ISOFORM per ‘GENE’	
Contig N10	773
Contig N20	573
Contig N30	460
Contig N40	384
Contig N50	329
Median contig length	272
Average contig length	334.57
Total assembled bases	24,885,474

### Transcriptome annotation

**Fig. 1:**
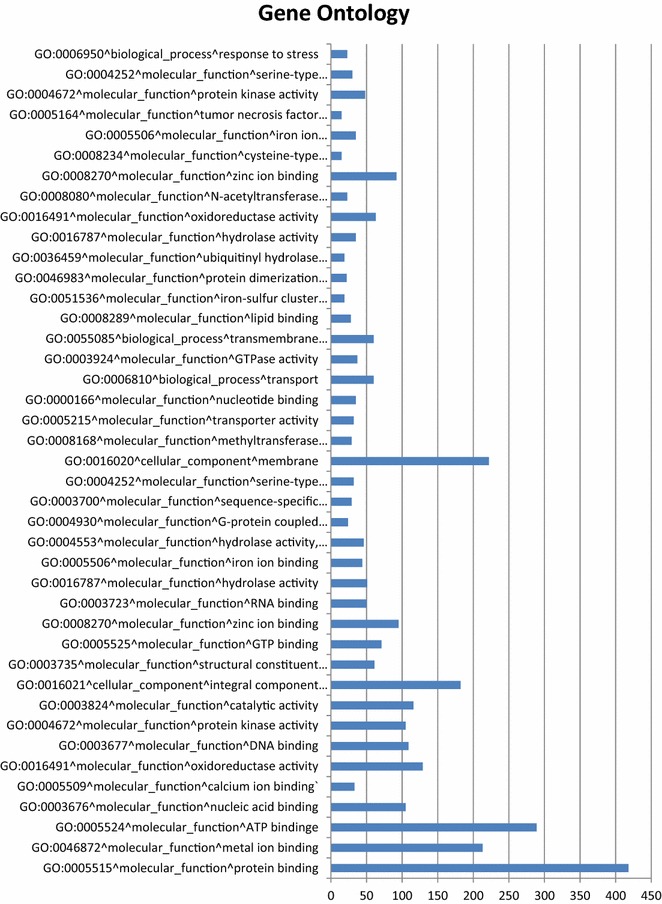
Analysis of transcriptome annotation by frequency of gene ontology description

Transdecoder 2.0.1 (http://transdecoder.sf.net) was used to identify candidate coding regions within the generated transcriptome and looked for an open reading frame of at least 100 amino acids long. This generated four output files (.pep peptide sequence for final candidate ORFs;.cds nucleotide sequence for coding regions;.gff3 position in target transcript of final ORFs and.bed format describing ORF that can be used in IGV). A BLASTP v2.3.0+ search against known proteins and HMMER 3.0 search to identify common protein domains were also carried out. Trinotate was used to carry out functional annotation of the transcriptome using the Trinity generated transcriptome file and the transdecoder generated peptide sequence file for final candidate ORFs, along with BLASTX v2.3.1, BLASTP v2.3.0+ and HMMER 3.0 searches and were loaded into an Sqlite database (see Additional file 1: Table S1). Figure 1 summarises the annotation data based on the classification by PFAM Gene Ontology (GO), the figure shows frequency of genes grouped on the basis of the GO classification that have a frequency in the annotated transcriptome of at least 15, the most frequent classification of genes were in the protein and metal binding category and cellular component membrane. Interestingly there were also over 30 hits with methyltransferases indicative that a potential DNA methylation mechanism could exist in the earthworm [13].

### Differential gene expression analysis

To identify differentially expressed transcripts; RSEM was first used to estimate expression values. Each of the six original.fasta raw sequence files (three adult and three juvenile pooled paired samples) were aligned to the de novo assembled Trinity.fasta transcript using Bowtie and RSEM used to estimate expression values based on the alignment. EdgeR was then used to calculate differential expression between the two conditions; with a P value cut off of 0.001, this returned six significantly differentially expressed genes, (most significant FDR) (Table 2). Initial BLASTX hits (Table 2) included hits with annelid ferritin and lysozyme proteins, as well as fungal NADH cytochrome b5 reductase and senescence associated proteins. At a cut off of P = 0.01 there were a further 26 differentially expressed genes.

**Table 2:** BLASTX v2.3.1 results of differentially expressed genes between adult and juvenile samples

Gene ID	P value and FDR	Sequence	BLASTX
TRINITY_DN212688_c0_g3_i1	5.70E−09 0.000322	CCTCAGTATGAGGATCCTCGTGCTTGATGGCCAACTTGTGCAATTCCAGCAAAGCCTCGTTAAGGCTCTTCTCCATATCCAGAGATGTCTGCATTGCTCCCAATGCGGTTCCCCAGGAGTCAACAGCCGGTTTCGAGACGTCGTCAAGAACAACGCAACCGCCACGCTTGTTCATGTACTTCATCATGTCACCAGCATGGCTTCTCTCTTCCTTTGAGTTTTCACTGAAGAACTTCGATAAGCTAGGCAGAGCCACTGAGTCATTATCAAAGTAGATCGCCATCGAATGAAAGTTGTAGGAAGCCTCAAGCTCCAGATTGATCTGCTTGTTCAGACACTTCTCAACCTCGACATGGAAGTTCTGACGAATCTGCGAGTCAGACAAAGATTC	Ferritin (*Eisenia andrei*)
TRINITY_DN228981_c22_g3_i1	1.15E−08 0.000322	TTTATCGTCTTTTTACTTATTCGCTGAAGCGGAGCTGGGTTTAACGACCCACCTTTTGGCTTTAAGGTCCTATACGGGCTGATCCGGGTTGAAGACATTGTCAGGTGGGGAGTTTGGCTGGGGCGGCACATCTGTTAAACCATAACGCAGGTGTCCTAAGGGGGACTCATGGAGAACAGAAATCTCCAGTAGAGCAAAAGGGCAAAAGTCCCCTTGATTTTGATTTTCAGTGTGAATACAAACCATGAAAG	Senescence associated protein (*Cennococcum geophilum*)
TRINITY_DN211598_c1_g4_i1	1.49E−08 0.000322	AGGTAAAGTTAACCAATGTAGTGGTGGCTACTGTGGACCGTATAAAATCTCGGAACCTTACTATAAGGACTGCGGAAAGCCAGGAGCCGGCTACCAACGATGTACTAAGCAGAAAGCTTGTTCGGAATTGTGCATCAAGTCTTACATGGAGCGCTACGCCACGAAATGTCCCAGACCTATTACCTGTGAAGACTATGCTCGCGTACACGTAGGAGGACCTGGCGGATGCACGAAGCCTTCAACCG	Lysozyme (*Eisenia fetida*)
TRINITY_DN203900_c3_g1_i1	4.58E−08 0.000741	GAAAATCCTAGGGAACGAATAATTTTCACGCCTGGTCGTACTCATAACCGCAGCAGGTCTCCAAGGTGAACAGCCTCTAGTTGATAGAACAATGTAGATAAGGGAAGTCGGCAAAATAGATCCGTAACTTCGGGATAAGGATTGGCTCTAAGGGTTGGGTGCGTTGGGCCTTGAGCAGAAGCCCTGGGAGCAGGTTGGCACTAGCCTCACGGCCGGCGCCTTCCAGCACCCGGTGGCGGACGCTCTTGGCAGGGTTCGCCCGTCCGGCGCACGCTTAACAACCAACTTAGAACTGGTACGGACAAGGGGAATCTGACTG	Hypthetical protein (*Tricoderma virens*)
TRINITY_DN218384_c10_g9_i1	8.03E−08 0.000948	GTTTCTTTTCCTCCGCTTATTGATATGCTTAAGTTCAGCGGGTATCCCTACCTGATCCGAGGTCAAAAGTGATAAATAATCTTGATGGATGCCAACAAAAATTGGTCTGTCACGCAAATTGTGCTGCGCTTCAAACCATTATATTAGCTGCCAATATATTTAAGGCGAGTCTAGGCTAAGCAAAGACAAACACCCAACACCAAGCAAGGCTTGAGAGTACAAATGACGCTCGAACAGGCATGCCCCCCGGAATACCAGGGGGCGCAATGTGCGTTCAAAGATTCGATGATTCACTGAATTCTGCAATTCACACTACTTATCGCATTTCGCTGCGTTCTTCATCGATGCCAGAACCAAGAGATCCGTTGTTGAAAGTTGTAATTATTAAATTTATTCAGACGCTGATTGAAAATAAAAAGGTTATAGAGTTGTCCAGCCGGCAGGCAAGCCTGCCGAGGAAACATGAGTGCGCAAAAAGTCAGGGGTTAAGAAAAGAGGCGTACTTCTTCTCAAGCAAGGCCCAAGAAGTTTCCGTCTCCCTCAAACAGTAGTAAACTACTGAAATTAATGATCCTTCCGCAGGTTCACCTACGGAAA	NADH cytochrome b5 reductase (*Aspergillus*)
TRINITY_DN13199_c0_g1_i1	8.80E−08 0.000948	CCCCGTTACCCGTTGATACCATGGTAGGCCACTATCCTACCATCGAAAGTTGATAGGGCAGAAATTTGAATGAACCATCGCCGGCGCAAGGCCATGCGATTCGTTAAGTTATTATGATTCACCAAGGAGCCCCGAAGGGCATTGGTTTTTTATCTAATAAATACACCCCTTCCGAAGTCGAGGTTTTTAGCATGTATTAGCTCTAGAATTACCACGGTTATCCAGGTAAAGGTACTATCAAATAAACGATA	Hypothetical protein (*Capitella teleta*)

## Discussion

Differential gene expression analysis was carried out on the de novo generated transcriptome to compare adult and juvenile samples. At a threshold of significance of P = 0.001 there were six significantly differentially expressed genes identified between adult and juvenile, potentially highlighting genes associated with sexual maturity for follow up in subsequent analyses with this and other earthworm species. Such targets may be important for investigating the impact of toxins on reproductive capability. The annotation of the transcriptome was dominated by protein and metal ion binding proteins, as expected, but there was also a suggestion of potential epigenetic mechanisms for gene control existing in *E. fetida* with methyltransferase function also occurring with relatively high frequency in the annotated transcript. This supports of the findings of Santoyo et al. [6] in that earthworms may be useful in experiments investigating the impact of terrestrial toxins on DNA methylation. From the perspective of genes involved specifically in reproduction, the annotation identified multiple sperm, spermatogenesis, testis, ova and other and reproductive associated proteins that could be potentially good targets for further studies exploring the impact of toxin on reproductive capability. Subsequent studies investigating a potential epigenome in *E. fetida* could also provide important data in terms of further understanding the relationship between environmental toxins and gene expression. Future work will involve using both transcriptome and epigenome analysis to explore the impact of the environment on gene expression and health in *E. fetida* and other earthworm species.

## Conclusions

This report illustrates the scope of what can be done with limited resources using Trinity and Trinotate freeware to assemble and annotate a RNA sequenced derived transcriptome de novo. The data generated have been made publicly available. To our knowledge this is the most comprehensive transcriptome for *E. fetida* and will provide an important baseline for further ecotoxicogenomic studies focusing on the impact of environmental toxins on global gene expression in *E. fetida* and other earthworm species.

## Additional file

**Additional file 1: Table S1.** Functional annotation of the Trinity generated transcriptome file.

## Abbreviations

EST: expressed sequence tags
RNA: ribonucleic acid
DNA: deoxyribonucleic acid
ORF: open reading frame
